# Early Adolescents and Substance Use

**DOI:** 10.1155/2013/307845

**Published:** 2012-08-27

**Authors:** Raimondo Maria Pavarin, Dario Consonni

**Affiliations:** ^1^Epidemiological Monitoring Center on Addiction, DSM-DP, ASL Bologna, Via S. Isaia 94/A, 40100 Bologna, Italy; ^2^Unit of Epidemiology, Department of Preventive Medicine, Fondazione IRCCS Ca' Granda-Ospedale Maggiore Policlinico, Via San Barnaba, 8-20122 Milan, Italy

## Abstract

1300 students (54.3% girls) 13–16 years old were interviewed in the urban area of Bologna during 2010. Random effect multiple logistic regression models were used. Results show a reciprocal relationship between alcohol use, tobacco, and cannabis. Most users were offered cannabis, began using at 14 years of age, and do not believe using is very dangerous. They live with only one parent, have more than 50 euros of spending money per month, and abuse alcohol, abuse that increases relative to the intensity of cigarette smoking. Legal/illegal dichotomy seems to overturn, where alcohol becomes a “drug” and the use of tobacco, similar to other drugs, is motivated as a solution to reduce anxiety, combat boredom, relax, and to ease loneliness.

## 1. Introduction

In Italy among 15 year olds, in the last year 9% have used cannabis at least once, 69% have used alcohol, and 34% have used tobacco, and this data increases at 16 years of age (17% cannabis, 80% alcohol, and 44% tobacco), with a greater prevalence of tobacco use among girls and alcohol and cannabis among boys [1]. Many try and then quit, but higher rates of continued use are evident for alcohol and tobacco [2]. 

Substance use in adolescence is an important predictor of possible continued use of illegal substances in adulthood, together with other risk factors: specific lifestyles outside the home (bar, discos, avd private parties), early start of sexual activity, a greater amount of spending money, frequenting urban environments or areas with a high prevalence and availability of illegal substances, the use of substances at home, family composition, and the development of various forms of sociability [3–9]. 

There is additional evidence that the decision to consume various substances is not connected only to specific contexts or individual characteristics, but that beliefs and expectations predict consumption styles. More recent studies are aimed at the decision-making processes of the adolescent where the possible costs and the potential expected benefits of consumption are considered [10–12]. In fact, given a set combination of experiences, abilities, information, and initiation of use, the choice of whether to use a substance followed by which to use, seems driven both by the function that it serves, as by the specific significances attributed to them by the consumer [13–22].

While most prevention programs are aimed at helping young people stay abstinent and to resist peer group pressure, there are few studies that describe the processes which drive the choice of utilizing diverse substances, studies that could help to develop and inform innovative approaches, especially in the education field and dissuasion efforts [17].

This study is aimed at identifying what drives early adolescents (13–16 years) to use substances. 

## 2. Methods

*Study Design and Participants*. A cross-sectional study design was used. The target was composed of subjects between the ages of 13 and 16 years, recruited middle schools (third year) and high schools (first two years) in the province of Bologna.

In each school, there is a teacher who serves as a health referent, to whom a copy of the study protocol was sent and to whom the methodology and goals were explained. 

At the participating schools, the interviewers, by appointment, met with the various classes, and after having explained to the students the goals and objectives of the study, they individually interviewed the young people in private who had obtained written permission from their parents. 

To the young people, in addition to the guarantee of anonymity and the confidentiality of the interview, they were guaranteed that the results would not be separated by age group, but considered as a total. The interviews, carried out from February to May 2010, lasted approximately 10 minutes on average. Four interviewers who were experts in interacting with early adolescent were utilized. 

Seven middle schools and two high schools participated. 

*Variables*. A semistructured interview was created to be utilized in this study. Twenty people were interviewed in succession by two interviewers. Kappa statistics [23] were used to verify the comprehensibility of the questions, the congruity of the answers, and the interviewer's effect. The variables used obtained a *K* value over 0.50. 

Variables utilized included demographic data (gender, age, domicile, and birth country); socioeconomic data (who do you live with, what grade, and monthly allowance); information about parents (occupational status); risk attributed to using various substances (score from 1 = *low* to 5 = *high*); use of substances in the last year (for each substance: age at first and last use, prevalent modality of use, modality of acquisition, and number of episodes of use); number of episodes of alcohol intoxication in the last year, and the CAGE test [24].

For each substance used, an open question was posed regarding the motive for use, and the responses were then codified into seven dichotomous variables (yes/no) after an analysis by a multidisciplinary team of experts including psychologists, psychiatrists, sociologists, and epidemiologists: to have fun and be with others, to improve sociability, curiosity, for pleasure, self-treatment for various types of malaise (anesthetic, analgesic, performance anxiety, and to alleviate sadness and depression), emulation, and to relax.

*Statistical Analyses*. Continuous and categorical variables were analyzed with Student's t and chi-squared test, respectively. To take into account possible correlation between students at each school, random effect multiple logistic regression models [25] were used to calculate odds ratios (ORs) with 95% confidence intervals (95% CI). All analyses were performed with Stata 11 [26].

## 3. Results

*Study Subjects*. A total of 1300 subjects were interviewed: 15.9% were 13 years old, 46.5% were 14 years old, 28.6% were 15 years old, and 8.9% were 16 years old. Slightly more than half were girls (54.3%), 8.9% were non-Italians. Just under half (48.8%) did not regularly receive an allowance, 43.5% received 50 euros or less each month, and 7.7% more than 50 euros (Table 1). Just more than one-third (36%) believed smoking cigarettes is not very dangerous, and 20% believed drinking alcohol is not very dangerous, 7% thought that cannabis is not very dangerous.

Regarding home life, 81.8% lived with both parents, 14.5% with their mother only, 1.3% with their father only, 2.5% with their mother and new partner, and 0.3% with their father and new partner. 

At least one parent of 18.2% of subjects did not work: 1.7% of fathers were retired, 0.6% unemployed; 14% of mothers were housewives, 1.6% unemployed, and 0.2% retired. 

*Substances*. Over the course of the last year, one subject in three smoked cigarettes, one in four drank alcohol, 14% were inebriated, 7% used illegal substances, and 48% were completely abstinent (Table 2).

Regarding illegal substances, 75 subjects used marijuana, 33 hashish, 6 hallucinogenic mushrooms, 3 cocaine, 3 ketamine green (all girls) 2 LSD (both boys), 1 “speed,” and 1 salvia divinorum.

Average monthly expenditure for alcohol was 39 euros (data from 91 subjects), 42 euros for tobacco (data from 171 subjects), and 44 euros for cannabis (data from 37 subjects).

*Alcohol*. Average age of first use was around 13 years (boys 12.9, girls 13.1) and lasted two years (boys 2.6, girls 2.4). Boys had a higher prevalence of use and a lower perception of risk. 

184 subjects were inebriated at least once in the last year, 3.7% at least five times, data that does not change based on gender.

With regard to the CAGE test, where there were no gender differences, 4% thought they should reduce their drinking, 2.6% experienced discomfort or feelings of guilt due to drinking habits, 1.5% drank alcohol at least once upon waking, and 1.3% were criticized for their drinking habits. 2.3% responded positive to at least two items on the test, 0.8% (10 subjects) to at least three.

*Tobacco*. The average age of first use was 13.4 years and lasted 2.1 years, with slight differences between boys and girls.

Girls had a higher prevalence of use and a lower perception of risk. 

8% smoked fewer than 5 cigarettes a day, 4% from 5 to 9 cigarettes a day, and 4% more than 9, with more intense use among girls (more than 5 cigarettes a day: girls 9%, boys 6%).

*Cannabis*. For both genders the average age of first use was 14.2 years and lasted 1.5 years, 5% consumed very few times, and 1% more than 15 times in the last year.

Males had a higher prevalence of use and a lower perception of risk. 

Regarding the modality of acquisition, to 61% of users it was offered, 34% used the same seller regularly, 6% acquired sporadically, and 5% sought out specific environments.

Subjects who used cannabis were younger on average when they started using tobacco (12.99 years, 95% CI 12.70–13.27) than those who did not use cannabis (13.46 years 95% CI 13.35–13.58, *P* = 0.0003).

*Risk Profiles*. To construct a profile of users of various substances, a multivariate analysis was carried out using logistic regression (Table 3). To take into account possible correlations between students at each school, random effect multiple logistic regression models were used to calculate odds ratios (ORs) and 95% confidence intervals (95% CI). The variables used in the model were gender, age, housing situation, parental occupational status, economic availability, perception of risk, and substance used (number of obs 1300).

Regarding alcohol use, the following users were highlighted: males, with more than 50 euros a month to spend, had a low perception of the dangers of alcohol, who used tobacco (increases with intensity) and cannabis. 

Regarding tobacco use, the following stands out: females, with both parents employed, who had a low perception of the dangers of tobacco, used alcohol and cannabis. Probability increased in subjects with recent episodes of inebriation. 

Regarding cannabis the following were highlighted: males, who lived with only one parent, who had more than 50 euros a month to spend, and a low perception of the dangers of cannabis, used tobacco (increases with intensity) and had recent episodes of inebriation. 

*Those Who Abstain*. The same analysis, repeated for those who abstain entirely, highlighted subjects who live with both parents (OR 1.36, 95% CI 1.0–1.86), had less than 50 euros to spend per month (OR 2.93, 95% CI 1.84–4.66), with only one parent employed (OR 1.94, 95% CI 1.39–2.72), and had a high perception of the dangers of alcohol (OR 1.44, 95% CI 1.11–1.87), tobacco (OR 1.95, 95% CI 1.41–2.71), and cannabis (OR 1.95, 95% CI 1.48–2.55).

*Motives of Use*. Tobacco use was motivated by curiosity (37%), pleasure (17%), emulation (10%), relax (7%), and self-treatment (7%); alcohol is associated with entertainment (30%), sociability (26%), curiosity (19%), and pleasure (15%); cannabis is used out of curiosity (48%), sociability (25%), pleasure (14%), and entertainment (11%).

Seven variables were constructed, derived from adding the motives of use of any substance (40% used out of curiosity, 22% to improve social relations, 21% to have fun, 19% for pleasure, 8% to emulate others, 6% to self-treat, and 6% for relaxation), and the probability of use was calculated of the various substances adjusting for gender and age. 

Considering values that were statistically significant at 95%, the use of any substance to emulate others and self-treat was more probable for those who smoked cigarettes; consumption for entertainment reasons was more probable for those who use alcohol; relaxation was a motivation for those who use alcohol or tobacco; increasing sociability was probable for who use alcohol or cannabis; for pleasure and for curiosity instead seemed to motivate use of all three different substances (Table 4).

## 4. Discussion

The results, which show a high use of tobacco and widespread alcohol abuse, indicate a relationship between use of substances, parental absence, a lot of spending money and low perception of risk. This is confirmed by the profiles of abstinent subjects, from families with one parent employed outside the house, with less than 50 euro available a month, and who had a high perception of the dangers of the various substances. 

The probability of using alcohol was higher for males, who had more money available, and increased as cigarette smoking increased. 

With regard to tobacco, the probability of recent use was higher among females, among subjects whose parents both work, and among those who used alcohol or cannabis. 

Most cannabis users were offered the drug, began using at 14 and were more likely to not believe it as very dangerous, lived with only one parent, had more than 50 euros spending money per month, abused alcohol, and use increased as cigarette smoking increased. It is notable that these subjects began smoking cigarettes before others did. 

With regard to motives of use, except for curiosity and pleasure-seeking, which seemed common to all substances, self-treatment and emulation appeared specific for tobacco users, and entertainment for users of alcohol, while use for relaxation affected both; improving social relations was more probable among those who used alcohol or cannabis. 

With regard to available spending money and use of the various substances, previous studies have found a relationship to smoking even small amounts of tobacco [27–29], an increase in use of illegal substances in relation to increasing monetary availability [30], and an increase in alcohol use related to increases in available spending money for leisure time activities [31]. Contrary to expectation, some studies have reported that adolescents from higher social classes presented a significantly higher percentage of alcohol and tobacco consumption than their counterparts from lower social classes, while others have reported a greater risk to start smoking cigarettes, marijuana, and drinking alcohol related to situations of socioeconomic disadvantage [32, 33].

The reciprocal relationship between use of various legal and illegal substances has been reported by recent studies, where the importance of this aspect has been presented regarding the planning of prevention strategies [34, 35]. 

With regard to risk attributed to the use of various substances, we found confirmation of studies of tobacco and marijuana, where the subjective perception of dangerous behavior seemed to exercise a protective function [36, 37].

With regard to family, a protective effect was shown for intact families (both father and mother), where particular attention was reported regarding adolescents in transition into new family structures [38, 39]. Parental control seemed initially to prevent marijuana use, but the effects weakened throughout adolescence [40].

## 5. Conclusions 

This study presents some objective limits that indicate prudence in generalizing the results: only subjects who obtained consent from their parents were interviewed and the information communicated in the interviews could have been influenced by various factors, including the situation and the location. Despite this, the results offer useful indications for future prevention projects specific to early adolescents. 

Family composition, available spending money, and risk perception seem to influence nonconsumption more than consumption, suggesting that they should be considered as protective factors that work together, to use as indicators of a serene environment, communication, and parental presence. In fact, when only one parent works, there was a low availability of spending money and a high perception of risk connected to use of any substance; there was a higher probability of finding subjects who were completely abstinent. 

A strong association was shown between the use of alcohol, tobacco, and cannabis, where we found that those who used a specific substance did not believe it to be very dangerous. Particularly, a relationship was reported between early use of tobacco and later use of cannabis, which seems to delineate a specific progression, warranting further study. With regard to money, greater availability seemed to play a role in the use of alcohol and cannabis, but not in the use of tobacco. While various substances were consumed for similar reasons, providing plausible explanations to the succession of use and poly use, we found emulation and self-treatment to be specific motivations for tobacco use and entertainment to be specific for alcohol use. 

Among early adolescents, the legal/illegal dichotomy seems to overturn, where alcohol loses its functions related to alimentation and social relationships to become a “drug” for all intents and purposes, and the use of tobacco, similar to other drugs, is motivated as a solution to reduce anxiety, combat boredom, relax, and to ease loneliness. 

## Figures and Tables

**Table 1:** Characteristics of interviewed subjects.

		Males (594)	Females (706)	Males %	Females %	*P*
Age (years)	13	97	110	16.3	15.6	0.48
	14	280	325	47.1	46.0	
	15	172	200	29.0	28.3	
	16	45	71	7.6	10.1	

Nationality	Italian	530	654	89.2	92.6	0.03
	Other	64	52	10.8	7.4	

Lives with	Both natural parents	501	556	84.3	78.8	0.04
	Only one parent	78	127	13.1	18.0	
	Stepfamily	15	22	2.5	3.1	

Do parents work?	Only one	110	127	18.5	18.0	0.81
	Both	484	579	81.5	82.0	

Monthly allowance	≤50 euros	266	300	44.8	42.5	0.68
	>50 euros	46	54	7.7	7.7	
	No allowance	282	352	49.9	47.5	

**Table 2:** Substances used in the last year.

		Males (594)	Females (706)	Total (1300)	Males %	Females %	Total %	*P*
Alcohol	Abstinent	424	544	968	74.4	77.1	74.5	0.05
	Alcohol, no inebriation	80	69	149	11.5	9.8	11.5	
	Inebriation	90	93	183	14.1	13.2	14.1	
	Low perception of risk	156	109	265	26.3	15.4	20.4	<0.001

Tobacco	Abstinent	419	478	897	70.5	67.7	69.0	0.25
	<5 cigarettes/day	137	166	303	23.1	23.5	23.3	
	≥5 cigarettes/day	38	62	100	6.4	8.8	7.7	
	Low perception of risk	230	235	465	38.7	33.3	35.8	0.12

Cannabis	Abstinent	547	668	1215	92.1	94.6	93.5	0.14
	<15 episodes	39	29	68	6.6	4.1	5.2	
	≥15 episodes	8	9	17	1.4	1.3	1.3	
	Low perception of risk	53	38	91	8.9	5.4	7.0	0.03

**Table 3:** Profile of subjects who used substances in the last year—random effect multiple logistic regression*.

	Alcohol**		Tobacco**		Cannabis**	
	OR	95% CI	OR	95% CI	OR	95% CI
Male	1.44	1.06–1.96	0.72	0.54–0.95	2.81	1.48–5.34
Lives with only one parent	1.12	0.75–1.66	1.03	0.71–1.50	2.44	1.21–4.92
Both parents work	1.23	0.80–1.89	2.01	1.32–3.05	1.81	0.64–5.06
Monthly allowance > 50 €	1.78	1.08–2.95	1.58	0.96–2.59	2.35	1.10–5.02
Low alcohol risk	1.92	1.35–2.74	0.74	0.51–1.07	0.68	0.33–1.39
Low tobacco risk	1.05	0.77–1.44	1.89	1.41–2.53	1.17	0.61–2.23
Low cannabis risk	1.27	0.71–2.26	1.53	0.85–2.75	14.20	6.68–30.18
Tobacco abstinent	1.00	—	1.00	—	1	
<5 cigarettes/day	3.08	2.24–4.24			2.88	1.27–6.50
≥5 cigarettes/day	14.38	7.86–26.29			23.31	9.49–57.27
Alcohol abstinent	—	—	1.00		1.00	—
Alcohol no inebriation			2.26	1.53–3.33	1.26	0.46–3.41
Alcohol inebriation			7.66	5.09–11.53	4.33	2.07–9.04
Use of cannabis						
No	1.00	—	1.00	—	—	—
Yes	2.74	1.41–5.31	5.31	2.59–10.86		

*Adjusted by age, **Number of obs 1300.

**Table 4:** Motives substances use—random effect multiple logistic regression model*.

*►*	Tobacco			Alcohol			Cannabis		
	Yes/no	OR	95% CI	Yes/no	OR	95% CI	Yes/no	OR	95% CI
Entertainment	77/324	1.45	0.87–2.42	111/221	76.12	30.12–192.41	31/54	1.76	0.98–3.18
Sociability	77/324	1.54	0.94–2.53	99/233	15.93	9.10–27.87	32/53	2.0	1.12–3.57
Curiosity	177/224	13.69	9.17–20.43	116/216	1.99	1.36–2.89	45/40	1.75	1.02–3.01
Pleasure	87/314	8.08	4.52–14.46	72/260	3.21	1.95–5.31	34/51	2.55	1.44–5.23
Emulation	40/361	15.85	4.40–39.20	24/308	1.64	0.84–3.20	4/81	0.41	0.13–1.27
Relax	31/370	47.59	6.28–360.50	23/309	3.35	1.43–7.84	7/78	1.02	0.40–2.64
Self-treatment	31/370	18.59	5.38–64.28	18/314	0.96	0.42–2.16	10/75	2.14	0.87–5.26

*Adjusted for gender and age.

## References

[1] http://www.epid.ifc.cnr.it/Espad/materiale.htm.

[2] EMCDDA Annual report on the state of the drugs problem in Europe 2010. http://www.emcdda.europa.eu.

[3] Latimer W, Zur J (2010). Epidemiologic trends of adolescent use of alcohol, tobacco, and other drugs. *Child and Adolescent Psychiatric Clinics of North America*.

[4] Tu AW, Ratner PA, Johnson JL (2008). Gender differences in the correlates of adolescents’ cannabis use. *Substance Use and Misuse*.

[5] Guxens M, Nebot M, Ariza C (2007). Age and sex differences in factors associated with the onset of cannabis use: a cohort study. *Drug and Alcohol Dependence*.

[6] Hayatbakhsh MR, Najman JM, Bor W, O’Callaghan MJ, Williams GM (2009). Multiple risk factor model predicting cannabis use and use disorders: a longitudinal study. *American Journal of Drug and Alcohol Abuse*.

[7] Nation M, Heflinger CA (2006). Risk factors for serious alcohol and drug use: the role of psychosocial variables in predicting the frequency of substance use among adolescents. *American Journal of Drug and Alcohol Abuse*.

[8] Sutherland I, Shepherd JP (2001). Social dimensions of adolescent substance use. *Addiction*.

[9] OEDT-Focus sulle droghe Misurare la prevalenza e l’incidenza del consumo di stupefacenti. http://www.emcdda.europa.eu/attachements.cfm/att_33481_IT_Dif03it.pdf.

[10] Parker H, Aldridge J, Measham F, Haynes P (1998). *Illegal Leisure: The Normalisation of Adolescent Recreational Drug Use*.

[11] Parker H, Williams L, Aldridge J (2002). The normalization of ’sensible’ recreational drug use: further evidence from the North West England longitudinal study. *Sociology*.

[12] Measham F, Shiner M (2009). The legacy of ’normalisation’: the role of classical and contemporary criminological theory in understanding young people’s drug use. *International Journal of Drug Policy*.

[13] Cox WM, Klinger E (1988). A motivational model of alcohol use. *Journal of Abnormal Psychology*.

[14] Kuntsche E, Knibbe R, Gmel G, Engels R (2005). Why do young people drink? A review of drinking motives. *Clinical Psychology Review*.

[15] van der Poel A, Rodenburg G, Dijkstra M, Stoele M, van de Mheen D (2009). Trends, motivations and settings of recreational cocaine use by adolescents and young adults in the Netherlands. *International Journal of Drug Policy*.

[16] Brunt TM, van Laar M, Niesink RJM, van den Brink W (2010). The relationship of quality and price of the psychostimulants cocaine and amphetamine with health care outcomes. *Drug and Alcohol Dependence*.

[17] Boys A, Marsden J, Strang J (2001). Understanding reasons for drug use amongst young people: a functional perspective. *Health Education Research*.

[18] Boys A, Marsden J (2003). Perceived functions predict intensity of use and problems in young polysubstance users. *Addiction*.

[19] Boys A, Marsden J, Griffiths P, Strang J (2000). Drug use functions predict cocaine-related problems in young people. *Drug and Alcohol Review*.

[20] Ahmadi J, Ghanizadeh A (2000). Motivations for use of opiates among addicts seeking treatment in shiraz. *Psychological Reports*.

[21] Lee CM, Neighbors C, Woods BA (2007). Marijuana motives: young adults’ reasons for using marijuana. *Addictive Behaviors*.

[22] Zvolensky MJ, Vujanovic AA, Bernstein A, Bonn-Miller MO, Marshall EC, Leyro TM (2007). Marijuana use motives: a confirmatory test and evaluation among young adult marijuana users. *Addictive Behaviors*.

[23] Armstrong BK, White E, Saracci R (1992). *Principles of Exposure Measurement in Epidemiology*.

[24] Bernadt MW, Mumford J, Taylor C (1982). Comparison of questionnaire and laboratory tests in the detection of excessive drinking and alcoholism. *Lancet*.

[25] Horton NJ (2008). Review of multilevel and longitudinal modeling using Stata, second edition, by Sophia Rabe-Hesketh and Anders Skrondal. *Stata Journal*.

[26] StataCorp Stata: release 11. statistical software.

[27] Van Reek J, Knibbe R, Van Iwaarden T (1993). Policy elements as predictors of smoking and drinking behaviour: the Dutch cohort study of secondary schoolchildren. *Health Policy*.

[28] Reddy-Jacobs C, Téllez-Rojo MM, Meneses-González F, Campuzano-Rincón J, Hernández-Ávila M (2006). Poverty, youth and consumption of tobacco in Mexico. *Salud Publica de Mexico*.

[29] Kostova D, Ross H, Blecher E, Markowitz S (2011). Is youth smoking responsive to cigarette prices? Evidence from low- and middle-income countries. *Tobacco Control*.

[30] Burrone MS, Bueno SM, de Costa ML, Enders J, Fernández RA, Vasters GP (2010). Análisis de la frecuencia de experimentación y consumo de drogas de alumnos de escuelas de nivel medio. *Revista Latino-Americana de Enfermagem*.

[31] Rahkonen O, Ahlstrom S (1989). Trends in drinking habits among Finnish youth from 1973 to 1987. *British Journal of Addiction*.

[32] Pratta EMM, Dos Santos MA (2007). Adolescence and the consumption of psychoactive substances: the impact of the socioeconomic status. *Revista Latino-Americana de Enfermagem*.

[33] Lemstra M, Bennett NR, Neudorf C (2008). A meta-analysis of marijuana and alcohol use by socio-economic status in adolescents aged 10—15 years. *Canadian Journal of Public Health*.

[34] García EG, Blasco BC, López RJ, Pol AP (2010). Study of the factors associated with substance use in adolescence using association rules. *Adicciones*.

[35] Wium-Andersen IK, Wium-Andersen MK, Becker U, Thomsen SF (2010). Predictors of age at onset of tobacco and cannabis use in Danish adolescents. *Clinical Respiratory Journal*.

[36] Doran N, Sanders PE, Bekman NM (2011). Mediating influences of negative affect and risk perception on the relationship between sensation seeking and adolescent cigarette smoking nicotine. *Nicotine & Tobacco Research*.

[37] Lopez-Quintero C, Neumark Y (2010). Effects of risk perception of marijuana use on marijuana use and intentions to use among adolescents in Bogotá, Colombia. *Drug and Alcohol Dependence*.

[38] Paxton RJ, Valois RF, Drane JW (2007). Is there a relationship between family structure and substance use among public middle school students?. *Journal of Child and Family Studies*.

[39] Hemovich V, Lac A, Crano WD (2011). Understanding early-onset drug and alcohol outcomes among youth: the role of family structure, social factors, and interpersonal perceptions of use. *Psychology, Health and Medicine*.

[40] Tang Z, Orwin RG (2009). Marijuana initiation among American youth and its risks as dynamic processes: prospective findings from a national longitudinal study. *Substance Use and Misuse*.

